# An *in vitro* method for the analysis of hemocyte-derived extracellular traps in shrimp

**DOI:** 10.1016/j.mex.2023.102220

**Published:** 2023-05-14

**Authors:** María Soledad Morales-Covarrubias, Blanca Alicia Ramírez-Azpilcueta, Jenny Antonia Rodríguez, Rafael Diego Rosa

**Affiliations:** a Centro de Investigación en Alimentación y Desarrollo A.C., Unidad Mazatlán en Acuicultura y Manejo Ambiental. Avenida Sábalo Cerritos s/n, Mazatlán, Sinaloa C.P. 82100, México; b Centro Nacional de Acuicultura e Investigaciones Marinas (CENAIM), Escuela Superior Politécnica del Litoral (ESPOL), Campus Gustavo Galindro Km 30.5 Vía Perimentral, P.O. Box 09-01-5863, Guayaquil, Ecuador; c Department of Cell Biology, Laboratory of Immunology Applied to Aquaculture, Embryology and Genetics, Federal University of Santa Catarina, Florianópolis 88040-900, Brazil

**Keywords:** Invertebrate immunity, Extracellular nucleic acids, Cellular immunity

## Abstract

The formation of extracellular traps (ETs) is a cell death mechanism relying on the release of nucleic acids in response to different stimuli. More recently, ETs have been recognized as an important cellular immune response since they are able to entrap and kill various microorganisms. The main goal was to describe a methodology to induce and visualize the *in vitro* formation of ETs by shrimp hemocytes. ETs formation was induced by the incubation of hemocyte monolayers from naïve shrimp (*Penaeus vannamei*) with a standard dose of *Vibrio parahaemolyticus* M0905. Following fixation, slides were stained with 4′,6-diamidino-2-phenylindole (DAPI) and imaged by fluorescence microscopy. The methodology proposed in this study successfully induced the formation and release of hemocyte-derived ETs in penaeid shrimp. The procedure described here can be used as a novel immune marker to assess shrimp health status.

## Graphical abstract

**Figure:**
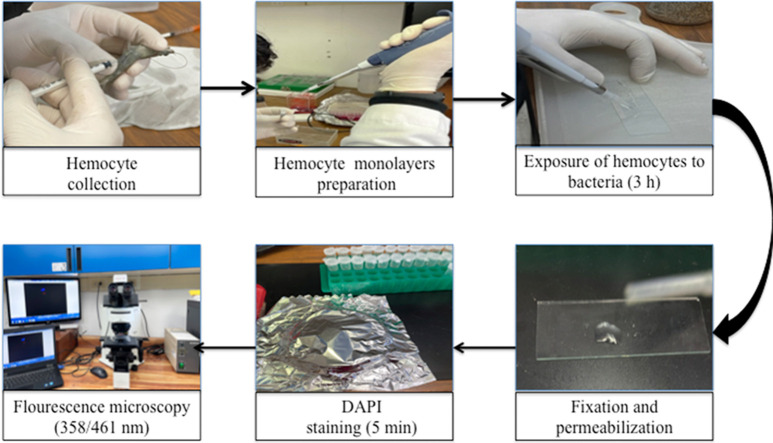

Specifications table

**Table:** 

Subject area:	Immunology and Microbiology

More specific subject area:	Shrimp immunology
Name of your protocol:	An *in vitro* method for the analysis of hemocyte-derived extracellular traps in shrimp
Reagents/tools:	Reagents: - Ethanol (70% [v/v] in distilled water) - Anticoagulant: Modified Alsever Solution (MAS: 27 mM sodium citrate, 336 mM NaCl, 115 mM, 9 mM EDTA, pH 7.0) - Formaldehyde (3.7% [w/v] in distilled water) - Triton X-100 (0.01% [v/v] in distilled water) - 10 × Phosphate-buffered saline (PBS: 1.37 M NaCl, 27 mM KCl, 100 mM Na_2_HPO_4_, 18 mM KH_2_PO_4_, pH 7.2) - DAPI stain solution (4′,6-diamidino-2-phenylindole; D9542-Sigma-Aldrich)
	Equipment: - Cotton swab - 1 mL insulin syringes - Conventional uncoated glass slides - Coverslip - Hemocytometer (Neubauer chamber) - Microtube centrifuges (1.5 mL) - Micropipettors (100, 200 and 1000 µL with corresponding tips) - Fluorescence microscopy
Experimental design:	We describe here a facile and low-cost procedure to induce the *in vitro* formation of ETs by shrimp hemocytes. In contact with a standard dose of the Gram-negative *Vibrio parahaemolyticus* M0905, circulating hemocytes are able to produce and release non-condensed DNA fibers that can be easily visualized by 4′,6-diamidino-2-phenylindole (DAPI) staining using a fluorescent microscope.
Ethics:	The shrimp for all the experimental work were used in accordance with the protocols of the Official Mexican Standard (NOM-062-ZOO-1999).
Value of the Protocol:	• A simple and reliable tool that requires few equipment resources to evaluate in short time (3 h) the formation and release of hemocyte-derived ETs in penaeid shrimp. • Compared to other methods for assessing shrimp health status, this low-cost procedure only requires DAPI staining. • Shrimp do not need to be sacrificed for the evaluation of this cellular immune parameter.
Trial registration (if applicable):	N.A.

## Description of protocol

Infectious diseases caused by viruses and pathogenic bacteria are undoubtedly the main factors that limit the shrimp farming industry worldwide. To defend themselves from invading microorganisms, penaeid shrimp rely on the combination of cellular and humoral responses mediated mainly by hemocytes, the circulating immunocompetent cells [1]. Microbial recognition by pattern recognition receptors leads to hemocyte mediated reactions, such as phagocytosis, nodule and capsule formation, tissue infiltration and the production of reactive oxygen species (ROS) and antimicrobial host defense peptides (AMPs) [2]. Additionally, a new cellular immune reaction, named extracellular traps (ETs), was also described in crustaceans, in which DNA networks associated to histones are released by hemocytes to entrap and kill bacteria [3,4].

The formation and release of ETs is a conserved antimicrobial process present from protozoa to multicellular organisms, including plants and both vertebrates and invertebrates [5]. Actually, ETs is a cell death mechanism dependent on the generation of ROS by NADPH oxidase [6], a phenomenon that was originally described in mammalian neutrophils [7]. ETs are mainly composed of chromatin-carrying histones associated with granular antimicrobial proteins and can be induced by either microbes (bacteria, fungi, parasites and viruses) or microbe-associated molecular patterns [8]. In penaeid shrimp, the extracellular DNA fibers released by circulating hemocytes are associated with the bactericidal c-type lysozyme protein and showed to be induced by phorbol myristate acetate (PMA), lipopolysaccharide (LPS) and peptidoglycan (PGN) as well as by live bacteria [3,9].

We describe here a facile and low-cost procedure to induce the *in vitro* formation of ETs by shrimp hemocytes. In contact with a standard dose of the Gram-negative *Vibrio parahaemolyticus* M0905, circulating hemocytes are able to produce and release non-condensed DNA fibers that can be easily visualized by 4′,6-diamidino-2-phenylindole (DAPI) staining using a fluorescent microscope. We propose the use of this technique as an additional cellular immune parameter in aquaculture to assess shrimp health status in different rearing or experimental conditions.

## Assay procedure

### Preparation of bacterial inoculum

1. Inoculate a bacterial colony in 10 mL of Tryptic Soy Broth (TSB) supplemented with 2% of NaCl (TSB+ Bioxon) in a 15 mL culture tube and shake at 30 °C for 24 h. In this study, we used the *Vibrio parahaemolyticu*s strain M0904 causing shrimp Acute Hepatopancreatic Necrosis Disease (AHPND), which was isolated from a shrimp farm affect by AHPND in northwestern Mexico.
2. Spin down the culture (2000 × g for 20 min at 20 °C) and resuspend the bacterial pellet with 2% NaCl until obtaining an optical density by ∼1.0 at a wavelength of 600 nm [1 × 10^8^ colony-forming units (CFU)/mL (0.5 MacFarland standard)].

### Hemocyte collection and counting

1. Load the 1 mL syringes with 100 µL of precooled Modified Alsever Solution (MAS).
2. Withdraw 100 µL of hemolymph (1:1; MAS:hemolymph) from the ventral sinus of shrimp (*Penaeus vannamei*), which is located at the basis of the first abdominal segment. To avoid contamination, clean the area with a cotton swab soaked in ethanol 70%.
3. Remove and discard the needle from the syringe and place the hemolymph sample into a precooled 1.5 mL microcentrifuge tube.
4. Mix hemolymph samples by gently pipetting up-and-down in order to prevent cell clumping.
5. Transfer 10 µL of the hemolymph sample onto a hemocytometer.
6. Using a light microscope, count and record the number of hemocytes in each one of the four outer squares of the hemocytometer (Neubauer chamber).
7. Each square in a hemocytometer with a coverslip placed represents 10^−4^ cm^3^ and 1 mL is equivalent to 1 cm^3^, thus the number of hemocytes/mL of hemolymph = average number of cells in all four squares x 2 dilution factor (1 MAS: 1 hemolymph) x 10^4^.
8. Adjust the dilution with MAS to 1 × 10^6^ cells/mL.

### Monolayers preparation and bacterial stimulation

1. For hemocyte monolayer preparation, pipette 100 µL of the hemocyte suspension containing 1 × 10^6^ cells/mL on a conventional uncoated glass slide and spread the cells uniformly using a pipette tip.
2. Incubate for 30 min at 25 °C to allow cell adhesion.
3. Remove any unattached cells by immersing the glass slide into a container filled with MAS for 1 min.
4. Drip the glass slide and pipette 100 µL of the bacterial inoculum containing 1 × 10^8^ CFU/mL of live *Vibrio parahaemolyticus* M0905.
5. Incubate for 3 h at 25 °C to allow hemocyte stimulation and the formation and release of ETs.
6. Remove the bacterial suspension by immersing the glass slide into a container filled with MAS for 1 min.

### DAPI staining protocol

1. Fix the cells by covering the glass slide with 3.7% formaldehyde in distilled water for 10 min at room temperature (RT).
2. Wash the cells 3 times with 1 × PBS for 5 min at RT.
3. Permeabilize cells with 0.01% Triton X-100 for 5 min at RT.
4. Wash the cells 3 times with 1 × PBS for 5 min at RT.
5. Add sufficient 300 nM DAPI stain solution to cover the cells.
6. Incubate for 5 min protected from light.
7. Remove the stain solution.
8. Wash the cells 3 times with 1 × PBS for 5 min at RT.
9. Place the cover slip. Keep slides in the dark from this step forward using slide box or similar.
10. Slides are now ready for fluorescence microscopy (excitation/emission at 358/461 nm).

## Method validation

**Fig. 1:**
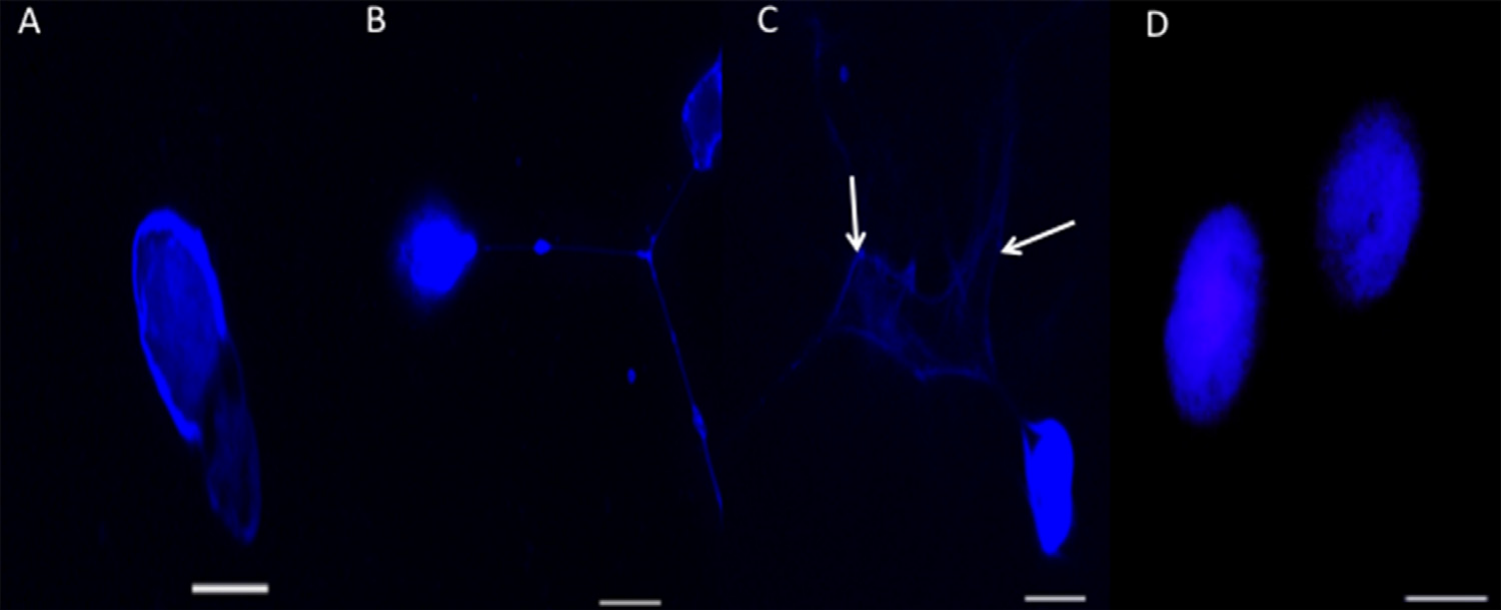
DAPI staining evidencing the *in vitro* formation of extracellular traps (ETs) by shrimp hemocytes (1 × 10^5^ cells) in contact with 1 × 10^7^ CFU of live *V. parahaemolyticus* M0905 at 1 h (A), 2 h (B) and 3 h (C). (D) DAPI staining showing the morphology of the nucleus of untreated (control) hemocytes. The white arrows show the bacterial cells entrapped by ETs. Scale bars = 30 µm.

We described here a methodology to induce the *in vitro* formation and release of ETs by the circulating hemocytes of penaeid shrimp (*P. vannamei*). The formation of ETs is an evolutionarily conserved cellular immune response relying on the release of nucleic acids in response to different stimuli [5]. In penaeid shrimp, the formation and release of DNA fibers by hemocytes (the immune cells) showed to be induced by microbial-associated molecular patterns (LPS, PGN) and by a non-pathogenic bacterium for crustaceans, *Escherichia coli* [3,9]. Here, we successfully induced the *in vitro* formation of hemocyte-derived ETs using the shrimp pathogen *V. parahaemolyticus* M0905. During the first hour of stimulation (100 CFU: 1 hemocyte), cells formed highly decondensed chromatin structures revealed by DAPI staining (Figs. 1 and S1). After 2 and 3 h, hemocyte-derived ETs showed the form of flexible fibers and meshes (Figs. 1 and S1). Besides, the released ETs were able to entrap the *Vibrio* cells within their fibers (Fig. 1).

Hemocyte-derived ETs appear to be a previous step for nodule and capsule formation. In this process, different hemocyte subpopulations would take part [4,10]. It has been pointed out that hyaline and semi-granular cells release chromatin [4], and suffer lysis and nuclear alterations [10]. In this context, hyaline hemocytes could start the ET process due to its ability to undergo respiratory bursts by producing ROS [4], while the presence of melanin in the capsules indicates that there is also the degranulation of granular hemocytes [11]. Additionally to melanin, this cellular immune response showed to be also associated with the c-type lysozyme [9], a bactericidal protein involved in shrimp immunity [12]. Since the c-type lysozyme is produced by granule-containing hemocytes [11,13], it is likely that those cell subsets are also involved in the formation and release of ETs.

This easy-to-use protocol can find applications in aquaculture from the perspectives of assessing shrimp health status. Indeed, the analysis of cellular (total and differential hemocyte counts, hemocyte phagocytic activity, anion superoxide production, percentage of apoptotic hemocytes) and humoral (total serum protein concentration, coagulation-time of the hemolymph, phenoloxidase activity, serum agglutination titer, serum antimicrobial activity) immune parameters and gene expression analysis has been extensively used to assess the immunocompetence of farmed species in different conditions [14], [15], [16]. For instance, the ability of circulating hemocytes to trigger this cellular response can be compared between animals submitted to an experimental condition (*e.g.*, biotic and/or abiotic stressors) and to a control condition [15,16]. In this case, the percentage of hemocyte-derived ETs can be calculated based on the count of a minimum of 100 DAPI-stained nuclei. Hemocyte monolayers incubated with 2% NaCl can be used as technical control (Fig. 1). From another point of view, many immunostimulants are offered in the aquaculture market, and their ability to induce ETs can be used as an extra parameter for evaluation of their possible *in vitro* efficacy, before performing *in vivo* tests. In conclusion, the evaluation of hemocyte-derived ETs emerges as a new immune marker associated to shrimp cellular immune defenses.

## CRediT authorship contribution statement

**María Soledad Morales-Covarrubias:** Conceptualization, Methodology, Visualization, Investigation, Data curation, Writing – original draft, Writing – review & editing. **Blanca Alicia Ramírez-Azpilcueta:** Data curation. **Jenny Antonia Rodríguez:** Writing – review & editing. **Rafael Diego Rosa:** Writing – review & editing.

## Declaration of Competing Interest

The authors declare that they have no known competing financial interests or personal relationships that could have appeared to influence the work reported in this paper.

## Data Availability

Data will be made available on request.

## Appendix Supplementary materials

Image, application 1
