# Supplementary material for: An in vitro method for the analysis of hemocyte-derived extracellular traps in shrimp
Source: MethodsX. 2023 May 14;10:102220. doi: 10.1016/j.mex.2023.102220 (PMC10205528; doi:10.1016/j.mex.2023.102220)
Supplement: Supplementary file 1 [file mmc1.docx]

**Supplementary material**


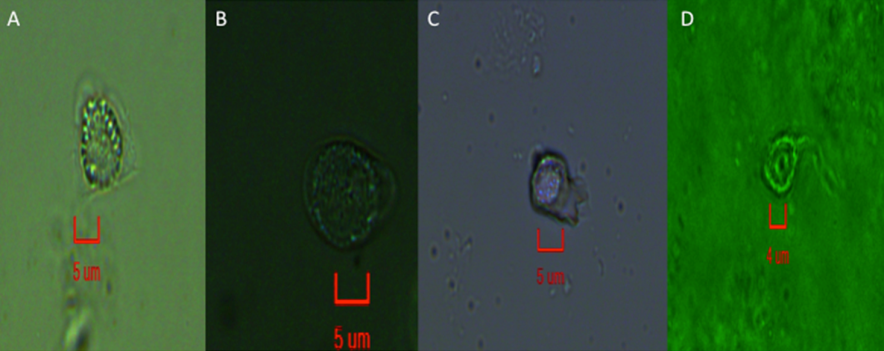


**Figure S1**. Images obtained by phase-contrast microscopy showing the *in vitro* formation of extracellular traps (ETs) by shrimp hemocytes (1×10^5^ cells) in contact with 1×10^7^ CFU of live *V. parahaemolyticus* M0905 at 0 h (A), 1 h (B), 2 h (C) and 3 h (D).
